# The Representation of Body Size: Variations With Viewpoint and Sex

**DOI:** 10.3389/fpsyg.2019.02805

**Published:** 2019-12-17

**Authors:** Sarah D’Amour, Laurence R. Harris

**Affiliations:** Department of Psychology, Centre for Vision Research, York University, Toronto, ON, Canada

**Keywords:** body representation, perceived size, full body perception, viewpoint, perceptual size distortions, height, body width

## Abstract

Perceived body size is a fundamental construct that reflects our knowledge of self and is important for all aspects of perception, yet how we perceive our bodies and how the body is represented in the brain is not yet fully understood. In order to understand how the brain perceives and represents the body, we need an objective method that is not vulnerable to affective or cognitive influences. Here, we achieve this by assessing the accuracy of full-body size perception using a novel psychophysical method that taps into the implicit body representation for determining perceived size. Participants were tested with life-size images of their body as seen from different viewpoints with the expectation that greater distortions would occur for unfamiliar views. The Body Shape Questionnaire was also administered. Using a two-alternative forced choice design, participants were sequentially shown two life-size images of their whole body dressed in a standardized tight-fitting outfit seen from the front, side, or back. In one image, the aspect ratio (with the horizontal or vertical dimension fixed) was varied using an adaptive staircase, while the other was undistorted. Participants reported which image most closely matched their own body size. The staircase honed in on the distorted image that was equally likely as the undistorted photo to be judged as matching their perception of themselves. From this, the perceived size of their internal body representation could be calculated. Underestimation of body width was found when the body was viewed from the front or back in both sexes. However, females, but not males, overestimated their width when the body was viewed from the side. Height was perceived accurately in all views. These findings reveal distortions in perceived size for healthy populations and show that both viewpoint and sex matter for the implicit body representation. Though the back view of one’s body is rarely–if ever–seen, perceptual distortions were the same as for the front view. This provides insight into how the brain might construct its representation of three-dimensional body shape.

## Introduction

The body is such an important part of our life – without it, we would not even exist. We use our body to present ourselves and to perceive and interact in the world. Knowledge about body posture, position, size, and structure are required to interpret and react to sensory information that is constantly being received and that may be coded relative to the body (Kopinska and Harris, 2003; Harris et al., 2015). Processing sensations and generating actions requires the brain to accurately map and represent the body and the body-in-space. However, the first-person perspective of the body is highly restricted, and the third-person perspective afforded by a mirror provides only a limited view. We cannot directly see our entire body in the same way that we can view the entirety of our hands, arms, and legs. However, it is the full three-dimensional body that is represented in the brain (Kammers et al., 2009; Longo and Haggard, 2012). How is the brain able to form such a representation of the body when it is not able to see it from multiple viewpoints? How accurate is its representation? The question then becomes focused on body perception when seen in unfamiliar views, such as from the side or back, to better understand how the implicit body representation is built up in the brain. We aim to answer these questions by assessing how accurate people are at judging their full body size when viewing their body from various viewpoints of which only the frontal view would be familiar. We used our novel psychophysical method that provides an implicit measure of the internal body representation (D’Amour and Harris, 2017). Our method involves a participant choosing which of two images is most like their own body and adjusting one of the images accordingly. It ends when both images (reference and distorted) are equally likely to be chosen, neither of which actually matches their body representation. The representation is calculated as being between these values.

Body size perception has typically been looked at in those suffering from eating disorders as distortions and disturbances of perceived body size and shape most obviously occur in these populations (Molinari, 1995; Probst et al., 1995; Gardner and Brown, 2014). Such studies have often tended to focus on measuring body image – how one feels about one’s body from a cognitive, emotional, and subjective view – rather than looking at how the brain internally maps and represents the body.

The objective of the current study was to examine perceived full body size accuracy to determine baseline values of how distorted the brain’s representation might be in a healthy, young populations of both males and females. The perceived width and height of the full body was measured as seen from three different body viewpoints in order to assess how the accuracy of perception changes when the image is presented in familiar and unfamiliar views. Previous studies have suggested both men and women tend to overestimate body width (e.g., Dolan et al., 1987; Stephen et al., 2018) and have emphasized the importance of baseline judgments in the healthy population (Sadibolova et al., 2019). However, until the introduction of virtual avatars, most studies have used smaller-than-life-size photographs, which confound absolute judgments with aspect ratio judgments and perhaps explain why perceived height, which requires the use of full-size images, has been neglected. Estimates of people’s perception of their height have tended to come from actions, such as ducking under barrier (Stefanucci and Geuss, 2012) which may not correspond to perceptual measures. In photographs height tends to be underestimated (Kato and Higashiyama, 1998). We hypothesized that there would be significant deviations from accurate in our healthy population, with the body being perceived as bigger and also as shorter than its actual size, with greater distortions for body width.

There is a trend in this area of research to use images of the body as seen from the front – corresponding to the view most commonly seen in the mirror. However, being overweight is most obvious in the profile view: a view which can only be imagined without a complex arrangement of mirrors. There is thus a potential for a richer source of information from judgments of the body seen in side view (Swami and Tovée, 2007; Cohen et al., 2015a,b). We therefore predicted that there would be a difference between viewpoints. Familiar views (as seen in a mirror) were expected to be more accurate than unfamiliar views (side and back views that rely on a person’s imagination to visualize), so that the front view would be the most accurate and the back and side views would be the least accurate.

Sex and body satisfaction were also assessed to see how these factors might impact perceived body size. Men and women show different patterns of perceived body distortion with women being more prone to judge themselves as fatter (Fallon and Rozin, 1985). This asymmetry may even have a basis in the differential roles of the cortical hemispheres in the representation of the body (Mohr et al., 2007). Differences related to both sex and body satisfaction were therefore anticipated, with females and those with higher levels of body dissatisfaction showing greater perceptual distortions. Previous studies looking at body size perception have tended to concentrate on females (e.g., Slade and Russell, 1973; Gleghorn et al., 1987; Thompson and Spana, 1988; Molinari, 1995; Cornelissen et al., 2017; but see Dolan et al., 1987; Craig and Caterson, 1990). Thus, there is a relative lack of knowledge about how males represent their bodies and whether they might also show distortions in size perception. Here, we included both males and females. While previous research has shown that perceptual body distortions occur more in those dissatisfied with their bodies (e.g., Cash and Deagle, 1997; Probst et al., 1998; Stice and Shaw, 2002; Hrabosky et al., 2009; Mohr et al., 2011; Sand et al., 2011; Cornelissen et al., 2013; Mai et al., 2015), these studies have also focused on clinical eating disorder populations with high levels of body dissatisfaction and have often overlooked the healthy population. Based on these previous findings, we thought that there would be differences between low and high body dissatisfaction groups. We expected to find greater distortions for those in the high body dissatisfaction group, especially for the width conditions than for those in the low body dissatisfaction group. We also predicted that there would be strong positive correlations between body dissatisfaction and perceived size distortions.

## Materials and Methods

### Participants

Thirty-seven participants (18 females and 19 males) took part in the experiment (mean age = 21.24 years, SD = 7.61; mean BMI = 23.75, SD = 4.09; mean weight = 68.94 kg, SD = 14.39 kg; mean height = 169.93 cm, SD = 7.52 cm; mean Body Shape Questionnaire (BSQ) = 85.27, SD = 33.15). They were recruited from the York University Undergraduate Research Participant Pool and received course credit for taking part in the study. The protocol was approved by the York Ethics Board. All subjects gave written informed consent in accordance with the Declaration of Helsinki.

### Materials/Stimuli

#### Body Dissatisfaction

The Body Shape Questionnaire (BSQ) (Cooper et al., 1987) is a 34-item self-report questionnaire that was developed to assess concerns about body shape and experiences of feeling fat that participants may have experienced within the previous month. The test was administered before the experiment began to obtain a measure of body dissatisfaction. Higher scores indicate higher levels of body dissatisfaction. Participants were divided into high and low groups defined as whether their scores were above or below the overall mean score.

#### Photographs

**Figure 1:**
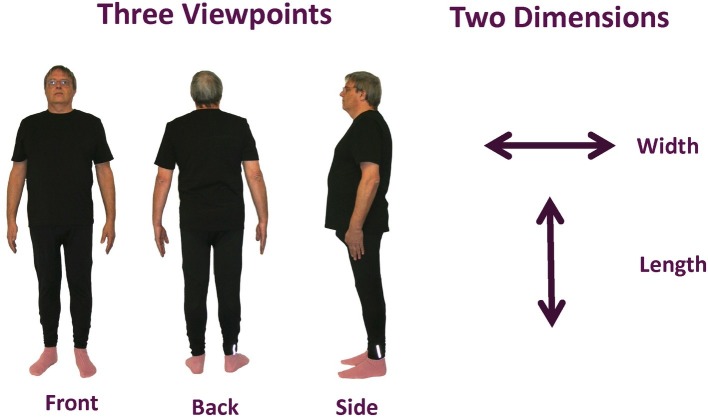
Experimental design and conditions. Sample images of the full body are shown for each viewpoint: front, side, and back. Width and length dimensions (indicated on the right of the figure) were distorted separately for each of the three viewpoints.

Color photographs of each participant’s whole body in standardized poses were taken using a digital camera (Canon EOS 10D; flash on; no zoom function) from each of three different viewpoints with a camera distance of 270 cm. Participants were asked to stand in front of a white wall in three standardized poses. Standardized outfits were provided to obtain accurate outlines of their size and shape (see Figure 1). The images were then corrected for any lens distortions, cropped to include only the whole body, and formatted on a white background (Adobe Photoshop CC 2014). These images served as the undistorted reference images and were used for composing distorted images. Actual body height was measured from the bottom of the feet to the top of the head using a ruler taped up to a wall. The image was presented life-size projected (using a BenQ 1080p short throw projector) onto a screen at a viewing distance of 270 cm by digitally adjusting the magnification of the image until it physically matched the participant’s actual body size. The viewing distance was chosen as matching the camera’s focal length multiplied by the magnification (Cooper et al., 2012), which minimizes distortions.

#### Distorting the Images

**Figure 2:**
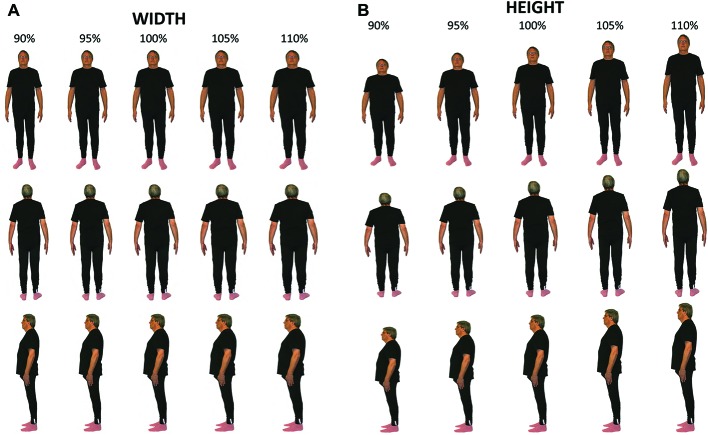
Examples of distorted images. Sample images of the distorted full body are shown for the **(A)** width and **(B)** height for each viewpoint - front, side, and back.

Images were presented and distorted using MATLAB (version 2011b) and Psychophysics Toolbox (Brainard, 1997) running on a MacBook Pro. One dimension of the image (either width – see Figure 2A – or length – see Figure 2B) was distorted (made either bigger or smaller) using a QUEST adaptive staircase psychometric procedure (Watson and Pelli, 1983). The image was viewed in the center of a projector screen with the full body shown from one of three viewpoints: (1) front, (2) side, or (3) back. Perceived width and height were measured separately for each viewpoint so there was a total of six experimental conditions.

### Procedure

Participants sat in a chair at a viewing distance of 270 cm from the projector screen. Each trial consisted of two 1.5 s intervals – one interval containing the undistorted image and one interval containing the distorted image presented in a random order – separated by a blank white screen for 1.5 s. Participants identified which interval contained the image that most closely matched their perception of their own body and responded using a two-button computer mouse (left button for first interval and right button for second interval). A QUEST adaptive staircase procedure (Watson and Pelli, 1983) was used with a two-alternative forced choice (2AFC) design to vary the chosen dimension (length or width) of the distorted image (D’Amour and Harris, 2017). Two interleaved QUEST staircases (25 trials per staircase) were used for each condition (50 trials total), with one starting with the manipulated dimension larger than natural and the other starting with that dimension smaller than natural. Each of the six conditions was run in a single block and took approximately 6 min to complete. Condition order was determined by a Latin square and was counterbalanced across participants.

### Data Analysis

The QUEST program returned an estimate of the percentage distortion relative to the undistorted at which the participant reported that the distorted image was as like their perceived body size as the undistorted image. The QUEST algorithm assumes the observer’s psychometric function follows a Weibull distribution and adaptively determines the amount of distortion to be presented based on the participant’s response to the previous trials. As the experiment goes on, knowledge on the observer’s psychometric accumulates. Participant’s decisions were plotted against the distortion used for each trial and fitted with a logistic (Equation 1) using the curve fitting toolbox in MATLAB.

(1) $$\text{Decision = 1/}\left(1+\exp\left(-\left(x-x_{0}\right)/b\right)\right)$$

where *x*_0_ is the distorted value that was equally likely to be judged as matching the observer’s size as the undistorted photograph, and *b* is an estimate of the slope of the function. The size of the internal body representation was taken as the point half way between *x*_0_ and the accurate size. We then subtracted 100% from this value to derive a difference-from-accurate score where positive numbers corresponded to an overestimate and negative numbers to an underestimate. The values so obtained for each participant for each condition were examined for outliers, defined as falling outside ±3 standard deviations from the mean. If a value fell outside this range (three participants—two females and one male), the complete dataset for that participant was removed.

One-sample *t*-tests were conducted for each condition to assess whether difference-from-accurate values significantly differed from zero (accurate). Mixed measures analyses of variances (ANOVAs) were used for statistical analyses, with alpha set at *p* < 0.05 and *post hoc* multiple comparisons were made using Bonferroni corrections. Pearson correlations were used to determine the relationship between body dissatisfaction and accuracy. Since we had predicted that there would be a specific direction for the correlations, one-tailed *p*’s were used.

## Results

### Full Body Size Accuracy

Table 1 summarizes the results of *t*-tests showing that the perceived width when seen from the front and side viewpoints were significantly different from accurate.

**Table 1:** One-sample *t*-tests comparing mean accuracy errors (percentage distortions) to accurate (zero distortion).

	*M* (SEM)	*t*(33)	*p*	95% CI
**Width**				
Front	−3.15 ± 1.32	−2.39	0.023^*^	[−5.84, −0.46]
Side	−0.83 ± 1.29	−0.64	0.524	[−3.45, 1.79]
Back	−2.85 ± 1.14	−2.49	0.018^*^	[−5.18, −0.52]
**Length**				
Front	−0.71 ± 0.87	−0.82	0.420	[−2.47, 1.05]
Side	−0.07 ± 0.87	−0.08	0.934	[−1.85, 1.71]
Back	0.34 ± 0.91	0.37	0.714	[−1.51, 2.18]

*SEM, standard error of the mean; CI, confidence interval*.

*N = 34*.

* *p < 0.05*.

### Full Body Size Accuracy: Width Dimension

**Figure 3:**
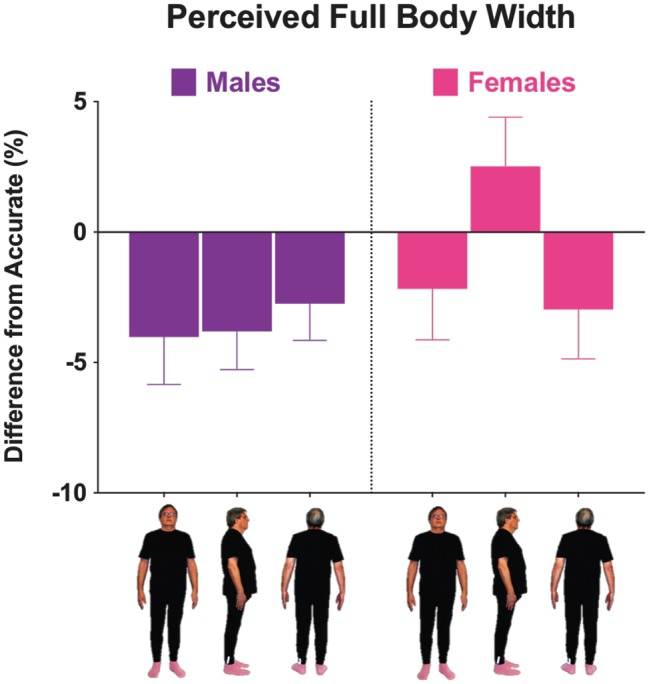
Mean differences from accurate for males (**left panel**) and females (**right panel**) when body width was distorted for each viewpoint. Positive and negative scores represent overestimation and underestimation, respectively. Error bars represent ±1 SEM.

A three-way mixed ANOVA was conducted to test for within-subject effects of viewpoint (front, side, and back), and between-subject effects of sex (male and female) and BSQ group (low and high) for the width dimension (Figure 3). A significant main effect of viewpoint, *F*(2, 60) = 3.38, *p* = 0.040, $\eta_{p}^{2}$ = 0.101, and a significant interaction between viewpoint and sex, *F*(2, 60) = 3.77, *p* = 0.028, $\eta_{p}^{2}$ = 0.112, were revealed. There was a difference in how width was perceived for the side view, with females showing greater overestimation from the side compared to both the front (*p* = 0.017) and back (*p* = 0.006) views with no significant difference between front and back views. Females’ side view estimates differed from male side view estimates (*p* = 0.019) with males underestimating their width in side view and females overestimating it. No interaction effects were found between viewpoint and BSQ group, *F*(2, 60) = 0.90, *p* = 0.413, $\eta_{p}^{2}$ = 0.029, or between viewpoint, sex, and BSQ group, *F*(2, 60) = 0.56, *p* = 0.576, $\eta_{p}^{2}$ = 0.018. There were no significant findings in any of the between-subjects effects tests.

### Full Body Size Accuracy: Length (Height) Dimension

**Figure 4:**
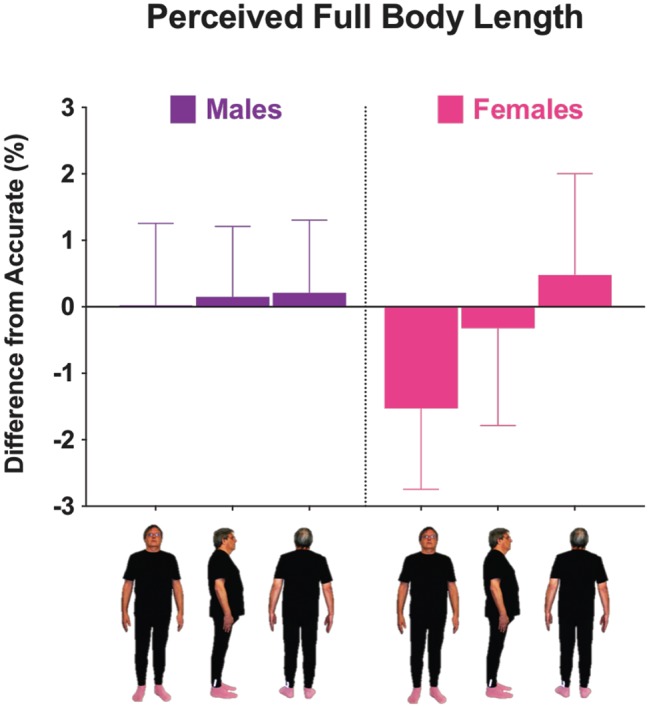
Mean differences from accurate for males (**left panel**) and females (**right panel**) when body length (height) was distorted for each viewpoint. Positive and negative scores represent overestimation and underestimation, respectively. Error bars represent ±1 SEM.

A second ANOVA was conducted using the same variables as above for the length (height) dimension (Figure 4). No significant main effects or interactions were found for the within-subjects effects tests. This suggests that perceived body length (height) was not impacted by seeing the body in different views. However, there was a significant interaction between sex and BSQ group, *F*(1, 30) = 7.51, *p* = 0.010, $\eta_{p}^{2}$ = 0.200. The high BSQ group differed (*p* = 0.026) in the distortion direction for males (overestimate: *M* = 2.71, SE = 1.72) and females (underestimate: *M* = −2.59, SE = 1.46). There were also non-significant trends when the high and low BSQ groups were compared for each sex (males: *p* = 0.075; females: *p* = 0.051).

### Correlations Between Perceived Full Body Size Accuracy and Body Shape Questionnaire Scores

**Figure 5:**
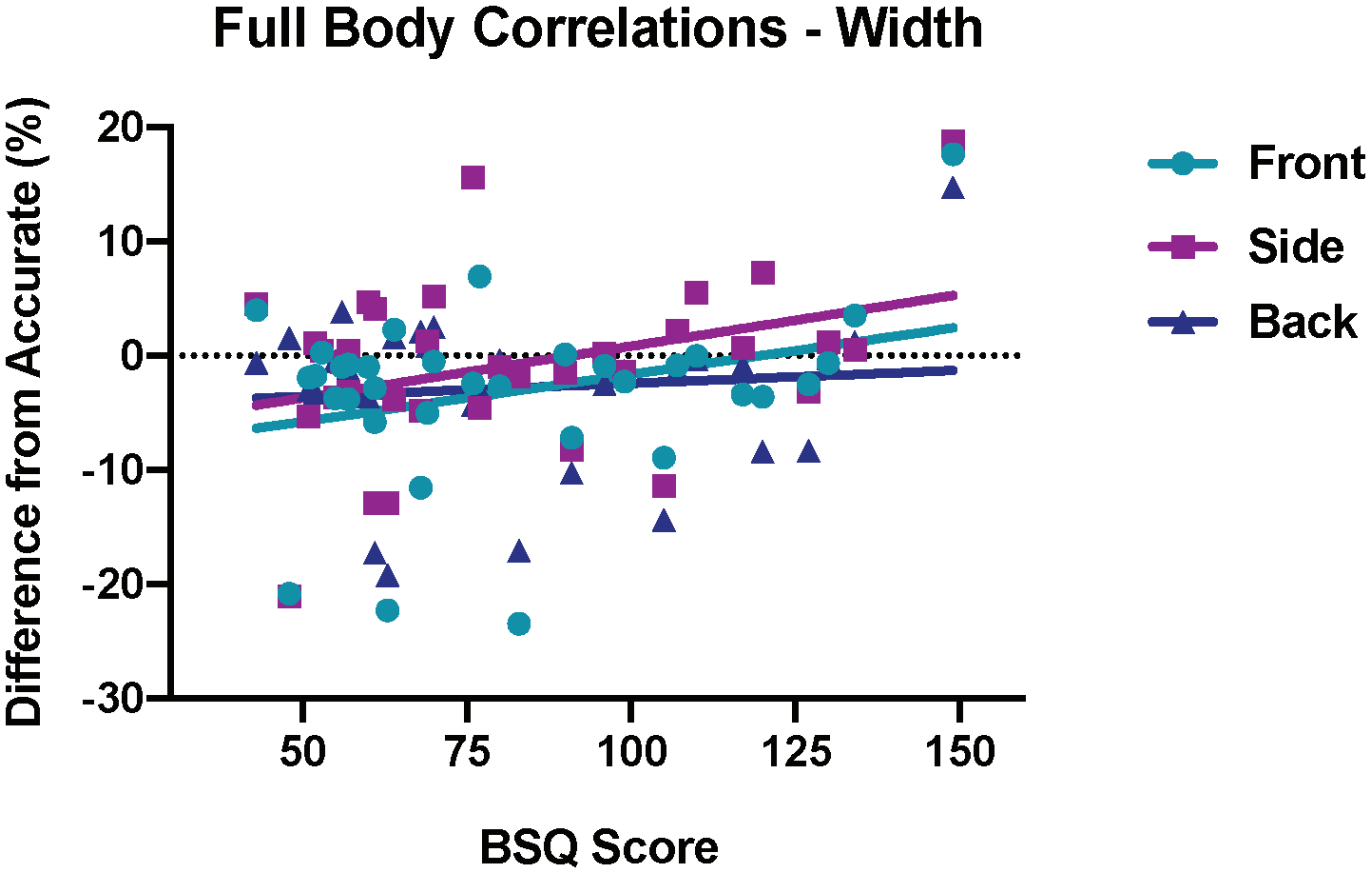
Correlations between BSQ score and differences from accurate for perceived body width for the front (blue circles), side (purple squares), and back (dark blue triangles) viewpoints (*n* = 34). The solid lines through the data represent linear regression fits.

Pearson correlations were run on the BSQ scores and differences-from-accurate to determine the relationship between body dissatisfaction and perceived size judgments. For the width dimension (Figure 5), there was a strong and significant correlation for the front view, *r*(33) = 0.310, *p* = 0.037, and the side view, *r*(33) = 0.349, *p* = 0.022, but no relationship was found for the back view, *r*(33) = 0.099, *p* = 0.289. There were no significant correlations between perceived size accuracy and BSQ score for the length (height) dimension [front: *r*(33) = −0.193, *p* = 0.138; side: *r*(33) = −0.077, *p* = 0.333; back: *r*(33) = −0.100, *p* = 0.287].

### Discussion

Width and length were measured for the full body from the front, side, and back view in order to obtain baseline accuracy values in a healthy population of males and females. We found that the full body was perceived as thinner (underestimating width) in the front and back views but when the body was viewed from the side, only females overestimated their width. A parallel can be found in emerging sex differences in hand perception where overestimation of hand width is larger in females (Coelho and Gonzalez, 2019; Longo, 2019). The height of the body was perceived as accurate. Our results reveal that viewpoint, sex, dimension (height/width), and body satisfaction matter for body representation. These findings provide insights into the mechanisms and factors that are involved in understanding how the body is processed, represented, and perceived.

### Overall Accuracy

Our finding that, independent of sex or body dissatisfaction, full body size was perceived as different from actual size when viewed from the front and side view when measured using a rigorous psychophysical method, is a novel finding that adds to the literature about body size accuracy in healthy populations. These results provide baseline measurements of distortions in full body perception at the level of the brain’s implicit body representation. The underestimations in body width that we observed have also been shown in some previous studies (e.g., Gardner et al., 1989). The finding that height was perceived accurately in all cases was unexpected because we make continual changes and adjustments to alter the height of our bodies at least as perceived by others such as by wearing heeled shoes, donning hats, and often by styling our hair. A unique feature of this study was that we used life-size photographs which are necessary to measure perceived height. While previous studies have looked at height estimation, they have typically used methods that require participants to make judgments based on apertures or barriers (e.g., Stefanucci and Geuss, 2012; Wignall et al., 2017), but these indirect measures cannot be applied to understanding the accuracy of the internal representation of body height.

### The Effect of Viewpoint

#### Front and Back View

Our predictions about the effects of viewpoint turned out to be the opposite of what we found. There was a general tendency to underestimate body width for males and females in accordance with Mazzurega et al.’s self-serving bias (Mazzurega et al., 2018). Interestingly, the front view (the view that we often see in a mirror) and the back (a view that we never see) showed the same distortions. And instead of familiar views being the most accurate, the front view actually showed the greatest amount of distortions. This may be a further support for the special relationship that the front and back of the body have with each other. The representations of the front and back of the body may be mapped together by the brain (Parsons and Shimojo, 1987; D’Amour and Harris, 2014; Harris et al., 2015; Hoover and Harris, 2015; Tamè et al., 2016). Thus, any distortion of one would be reflected in a comparable distortion of the other (see Figure 3).

#### Side View

Females perceived themselves to be wider than actual size only in the side view reminiscent of the female-only “fatter bias” of Mohr et al. (2007). There are several possible reasons for this. The side view is rarely if ever seen and therefore is most demanding on the viewer’s ability to visualize this view using only their internal representation. It may therefore be the best view with which to measure the size of this representation (Cohen et al., 2015a) and the one most able to reveal true distortions. We confirmed that, in this view, women are more likely than men to see themselves as fatter (Fallon and Rozin, 1985), but why might this be the case? Could this be due to the structure and functionality of a woman’s body? We did not ask whether any of our participants had been through pregnancy, and their youthfulness suggests that it would have been rare, but the potential for pregnancy involves an explicit expectation of flexibility in this front/back dimension (Franchak and Adolph, 2014). We speculate that this flexibility and the expectation of future expansion in this dimension, not expected by men, may underlie this sex difference. Another possible explanation is that females may have acquired a general tendency to see themselves as fatter than they really are – an illusion encouraged by any amount of advertising campaigns and the media (Thompson and Mikellidou, 2011; Docteur et al., 2012; Shin and Baytar, 2013; Gledhill et al., 2019). Hashimoto and Iriki (2013) found that slightly slimmer body images were most desirable as own-body images and that this tendency is most pronounced in women (Cazzato et al., 2012).

Another study (Cornelissen et al., 2018) aimed to determine which orientation was best for body size estimation tasks responding to the lack of research on how different viewpoints affect accuracy in body mass judgments. Since the majority of research has only presented the body from the front view, it is unclear whether this is the optimal viewpoint or if important visual cues that people use for size judgments are being obscured, such as stomach depth (Tovée et al., 1999; Smith et al., 2007; Rilling et al., 2009) and thickness of the thighs and buttocks (Cornelissen et al., 2009, 2016; Cohen et al., 2015a,b). While their study used computer-generated generic images and did not ask for own-body size judgments, they found a loss in precision for front view stimuli compared to both three-quarter and side views (Cornelissen et al., 2018) which supports our current findings.

### Sex and Body Satisfaction Scores

We have shown that distortions exist in both sexes for both low and high body dissatisfaction groups. Although there was surprisingly no effect of BSQ group on perceived width, there was a difference for perceived height between the males and females that were more dissatisfied with their bodies. On average across all three viewpoints, males in the high BSQ group perceived an increase in height, whereas females perceived a decrease. This finding could be due to attitudinal and societal factors that are experienced by each sex. When the relationship between BSQ score and perceived size accuracy was examined, it was revealed that higher body dissatisfaction showed greater distortions in perceived width for the front and side views. This is in agreement with Mazzurega et al. (2018) who related such findings to body attractiveness and what they called the self-serving bias. This bias is weaker in people who are less satisfied with their body and may result in greater distortions. It is difficult to compare our findings with previous studies since we used a population of healthy males and females and therefore had a much smaller range of BSQ scores than would be seen in females with eating disorders. Another potential limitation is that our sample size was quite small for conducting correlations and that we had an unequal amount of people in the low and high BSQ groups.

## Conclusion

Our results are important because they assess the internal representation of body dimensions independent of distortions of the body image. To extend our study and further the research done to gain knowledge about how the brain represents the body, future studies using 3D full body images/avatars should be done with our method to obtain more details about the brain’s modeling and mapping of body size, shape, and structure. Other potential research that could be beneficial for comparing and contrasting with our findings (and all previous literature) would be to use our method in different experimental designs, such as testing the effects of image size, distorting both dimensions at once, distorting only particular parts of the full body, or testing a greater range of viewpoints. Findings from such lines of research could be used to develop programs to retune body representations not only in clinical populations but also for athletes and dancers where accurate body representation is particularly critical.

## Data Availability Statement

The datasets generated for this study are available on request to the corresponding author.

## Ethics Statement

The studies involving human participants were reviewed and approved by the York Ethics Board. The patients/participants provided their written informed consent to participate in this study. Written informed consent was obtained from the individual(s) for the publication of any potentially identifiable images or data included in this article.

## Author Contributions

SD’A conceived the study and designed the experimental methodology. SD’A and LH devised and created the experimental methods, stimuli, and programming. SD’A performed the experiment and collected the data. SD’A analyzed the data and drafted the manuscript. Both authors contributed to the writing of the paper.

### Conflict of Interest

The authors declare that the research was conducted in the absence of any commercial or financial relationships that could be construed as a potential conflict of interest.
